# Superoxide Anions and NO in the Paraventricular Nucleus Modulate the Cardiac Sympathetic Afferent Reflex in Obese Rats

**DOI:** 10.3390/ijms19010059

**Published:** 2017-12-27

**Authors:** Qing-Bo Lu, Jing Sun, Ying Kang, Hai-Jian Sun, Hui-Shan Wang, Yuan Wang, Guo-Qing Zhu, Ye-Bo Zhou

**Affiliations:** 1 Key Laboratory of Cardiovascular Disease and Molecular Intervention, Department of Physiology, Nanjing Medical University, Nanjing 210029, China; 220163051@seu.edu.cn (Q.-B.L.); sun0704@njmu.edu.cn (J.S.); kangying@njmu.edu.cn (Y.K.); haijsunjiangnan@jiangnan.edu.cn (H.-J.S.); gqzhucn@njmu.edu.cn (G.-Q.Z.); 2 Department of Pediatrics, The Fourth Clinical Medical College of Nanjing Medical University, Nanjing 210029, China; wanghuishan@njmu.edu.cn (H.-S.W.); wangyuan@njmu.edu.cn (Y.W.)

**Keywords:** superoxide anions, nitric oxide, cardiac sympathetic afferent reflex, paraventricular nucleus, obesity

## Abstract

This study was conducted to explore the hypothesis that the endogenous superoxide anions (O_2_^−^) and nitric oxide (NO) system of the paraventricular nucleus (PVN) regulates the cardiac sympathetic afferent reflex (CSAR) contributing to sympathoexcitation in obese rats induced by a high-fat diet (42% kcal as fat) for 12 weeks. CSAR was evaluated by monitoring the changes of renal sympathetic nerve activity (RSNA) and the mean arterial pressure (MAP) responses to the epicardial application of capsaicin (CAP) in anaesthetized rats. In obese rats with hypertension (OH group) or without hypertension (OB group), the levels of PVN O_2_^−^, angiotensinII (Ang II), Ang II type 1 receptor (AT1R), and nicotinamide adenine dinucleotide phosphate (NADPH) oxidase were elevated, whereas neural NO synthase (nNOS) and NO were significantly reduced. Moreover, CSAR was markedly enhanced, which promoted the elevation of plasma norepinephrine levels. The enhanced CSAR was attenuated by PVN application of the superoxide scavenger polyethylene glycol-superoxide dismutase (PEG-SOD) and the NO donor sodium nitroprusside (SNP), and was strengthened by the superoxide dismutase inhibitor diethyldithiocarbamic acid (DETC) and the nNOS inhibitor N(ω)-propyl-l-arginine hydrochloride (PLA); conversely, there was a smaller CSAR response to PLA or SNP in rats that received a low-fat (12% kcal) diet. Furthermore, PVN pretreatment with the AT1R antagonist losartan or with PEG-SOD, but not SNP, abolished Ang II-induced CSAR enhancement. These findings suggest that obesity alters the PVN O_2_^−^ and NO system that modulates CSAR and promotes sympathoexcitation.

## 1. Introduction

The prevalence of obesity is increasing globally, and it is well known that obesity is often associated with hypertension and cardiovascular diseases (CVD). In addition, the activation of the sympathetic nervous system plays an important role in the pathogenesis of obesity-related hypertension and CVD [1,2,3,4]. 

The cardiac sympathetic afferent reflex (CSAR) induced by the stimulation of cardiac sympathetic afferents increases the sympathetic outflow and blood pressure (BP) in normal animals, which can cause a significant increase in cardiac contractility and cardiac output [5]. Our previous studies showed that the persistent enhancement of cardiac sympathetic afferent reflex (CSAR) promoted sympathetic overdrive in animal models [6,7,8,9]. The paraventricular nucleus (PVN) of the hypothalamus is not only an important integrative site in regulating the autonomic and cardiovascular activities [10,11], but also one of the main components of the central neurocircuitry of CSAR [12,13]. In addition, the PVN also participates in sympathetic overactivation in obesity and obesity-related hypertensive rats [14]. Thus, it can be supposed that the PVN is a major site in the regulation of CSAR in obese rats.

A number of studies indicated that oxidative stress is a key event during obesity that occurs not only at the level of the heart and blood vessels, but also within the central nervous system (CNS) [15,16,17]. The effects of central oxidative stress on the sympathetic regulation of BP have been exploited in some types of hypertensive animal models [18,19]. Moreover, the blockade of PVN superoxide anions (O_2_^−^) reduced the increased sympathetic nerve activity (SNA) in rats with heart failure or hypertension [18,19]. Accumulating evidence has suggested the association of obesity with angiotensin II (Ang II), which acts via the Ang II type 1 receptor (AT1R) and has many effects on cardiovascular diseases. The activation of the Ang II–AT1R axis plays a destructive role in the glucose and lipid metabolism, oxidative stress, inflammation, and insulin resistance during the pathogenesis of obesity [20]. It is established that the upregulated AT1 receptors in the PVN contribute to neuroinflammation in high-fat diet (HFD)-induced obesity in mice [21]. Ang II-induced O_2_^−^ generation via AT1R in the PVN has a key role in the modulation of the sympathetic outflow and the cardiovascular function [19,20], which is also associated with elevated nicotinamide adenine dinucleotide phosphate (NADPH) oxidase activity, a primary source of O_2_^−^ [20]. However, the importance of Ang II in the PVN in SNA is not completely understood in obesity.

Besides superoxide, nitric oxide (NO) is also involved in the regulation of SNA and BP [21,22]. Several studies have shown that NO inhibits SNA in the brain stem or the hypothalamus, and influences cardiovascular functions through sympathetic actions in the PVN [21,22]. The inhibition of neural NO synthase (nNOS) in the PVN elevates the mean arterial pressure (MAP), whereas increasing NO content in the PVN produces depressor responses in normal rats. Moreover, the downregulation of PVN NO was implicated in sympathetic overdrive in rats with hypertension and chronic heart failure (CHF) [5,22,23,24]. However, the association of O_2_^−^ and NO with CSAR and SNA in obesity has not been evaluated yet. In this study, we aimed to explore whether both O_2_^−^ and NO within the PVN contribute to the enhancement of CSAR and sympathoexcitation in high-fat diet (HFD)-induced obesity in rats.

## 2. Results

### 2.1. Metabolic and Anatomic Data, Blood Pressure (BP), and Heart Rate

After 12 weeks of HFD consumption, plasma insulin, cholesterol, and triglyceride levels, as well as heart weight, body weight, and white adipose tissue (WAT) mass were increased in obese rats without hypertension (OB) and obese rats with hypertension (OH). The systolic blood pressure (SBP), mean artery pressure (MAP), and heart rate (HR) in the OH group were significantly higher than in the other three groups (Table S1). 

### 2.2. Cardiac Sympathetic Afferent Reflex (CSAR)

**Figure 1:**
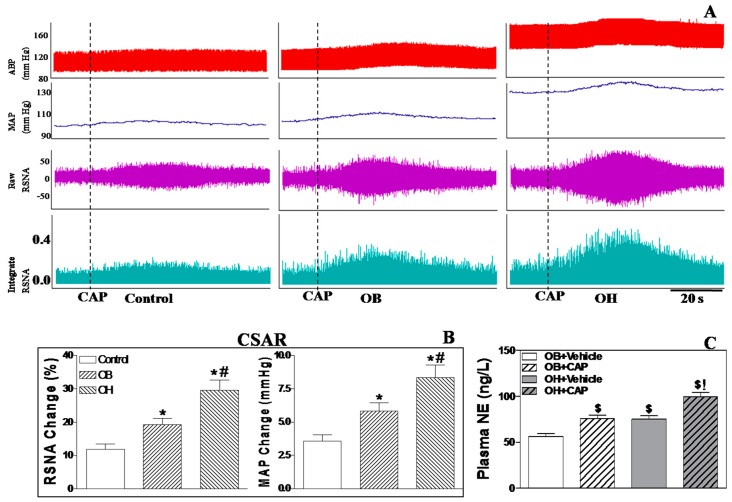
Representative recordings of the CAP-induced CSAR are shown by the RSNA and MAP responses to the epicardial application of CAP (**A**); the effect of the epicardial application of CAP on RSNA and MAP in control, OB, and OH rats (**B**); the effect of the epicardial application of CAP on plasma (NE) levels in OB, and OH rats (**C**). For each group, *n* = 6. The values are mean ± SE. * *p* < 0.05 versus control; # *p* < 0.05 versus OB; $ *p* < 0.05 versus OB+vehicle; ! *p* < 0.05 versus OH+vehicle. CAP: capsaicin; CSAR: cardiac sympathetic afferent reflex; ABP: arterial blood pressure; MAP: mean arterial pressure; RSNA: renal sympathetic nerve activity; OR: obesity resistant; OB: obesity without hypertension; OH: obesity-related hypertension; NE: norepinephrine.

Representative recordings of capsaicin (CAP)-induced CSAR are shown in Figure 1A. Epicardial application of CAP in the left ventricle increased renal sympathetic nerve activity (RSNA) and MAP in all four groups. The CAP-induced CSAR was much stronger in OH rats compared with OB rats (*p* < 0.05 for each, Figure 1B), and in OB rats compared with control and OR (obesity resistant) rats (*p* < 0.05 for each, Figure 1B). CSAR induction significantly increased plasma norepinephrine (NE) levels in OB and OH rats, and NE levels were much higher in OH rats than in OB rats (*p* < 0.05, Figure 1C). 

### 2.3. Microinjection of Polyethylene Glycol-Superoxide Dismutase (PEG-SOD) into the Paraventricular Nucleus (PVN)

**Figure 2:**
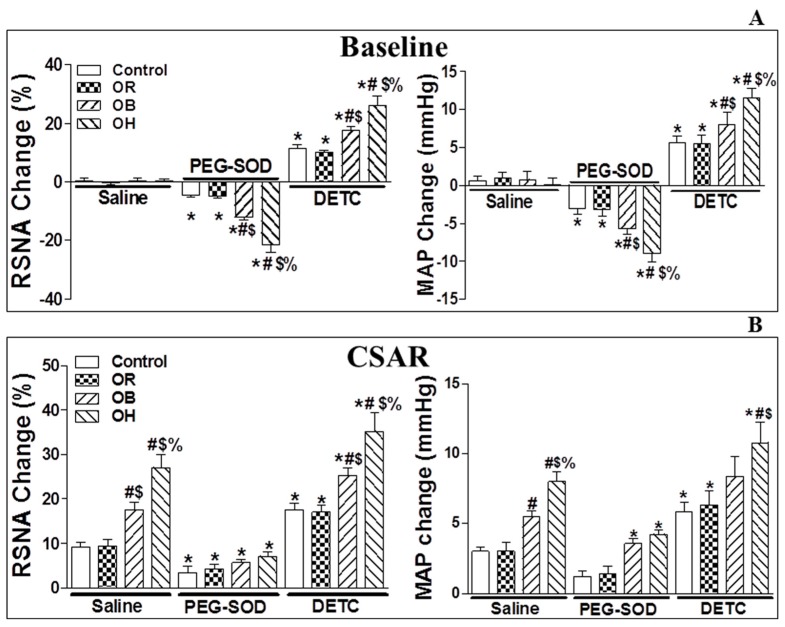
The effects of PVN microinjection of saline, polyethylene glycol-superoxide dismutase (PEG-SOD) (5 units) and diethyldithiocarbamic acid (DETC, 10 nmol) on the baseline RSNA and MAP (**A**), and on CSAR (**B**) in the PVN in control, OR, OB, and OH rats. The values are mean ± SE. For each group, *n* = 6; * *p* < 0.05 versus saline; # *p* < 0.05 versus control; $ *p* < 0.05 versus OR; % *p* < 0.05 versus OB. PVN: paraventricular nucleus; CSAR: cardiac sympathetic afferent reflex; RSNA: renal sympathetic nerve activity; MAP: mean artery pressure; OB: obesity without hypertension; OH: obesity-related hypertension; DETC: superoxide dismutase inhibitor.

Acute microinjection of PEG-SOD (5 units, an analogue of endogenous superoxide dismutase for scavenging O_2_^−^) into the bilateral PVN significantly lowered basal SNA (Figure 2A; the changes relative to baseline RSNA and MAP are shown in Table S2) and CSAR in the four groups compared with PVN microinjection of saline (*p* < 0.05 for all comparisons, Figure 2B). The peak of the depressor and sympathoinhibitory responses due to the microinjection of PEG-SOD into the PVN occurred around 20–30 min after the injection, so CSAR induction was started 20 min after PEG-SOD infusion. The duration of PEG-SOD action in the PVN was around 50–60 min. 

### 2.4. Microinjection of Diethyldithiocarbamic Acid (DETC) into the PVN

The infusion of diethyldithiocarbamic acid (DETC, 10 nmol, a superoxide dismutase inhibitor) into the bilateral PVN significantly increased basal SNA (Figure 2A) and CSAR in the four groups compared with PVN microinjection of saline, and these effects were much stronger in the OB and OH groups than in the control and OR groups (*p* < 0.05 for each, Figure 2B). The peak of pressor and sympathoexcitatory responses due to the microinjection of DETC into the PVN was around 4–10 min after the injection, so CSAR induction was started 5 min after DETC infusion. The duration of DETC action in the PVN was around 20–30 min. 

### 2.5. Microinjection of N(ω)-Propyl-l-Arginine Hydrochloride (PLA) into the PVN

**Figure 3:**
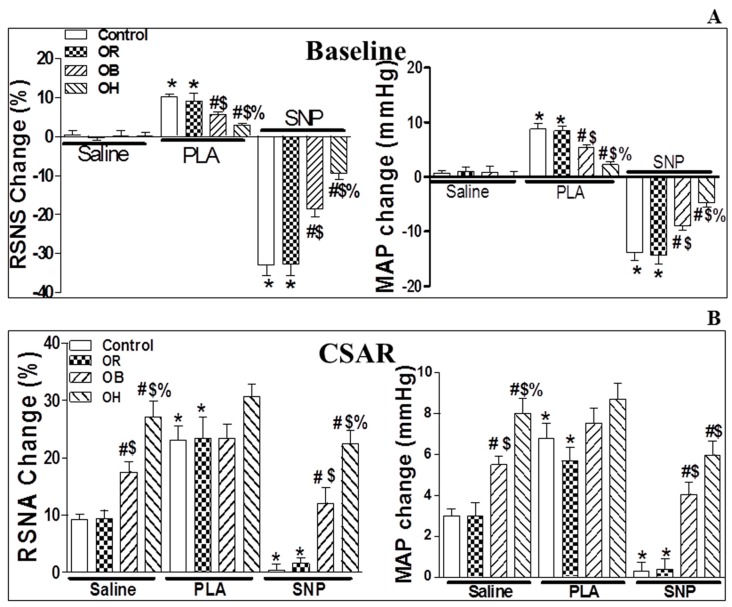
The Effects of PVN microinjection of N(ω)-propyl-l-arginine hydrochloride (PLA, 5 nmol) and sodium nitroprusside (SNP, 50 nmol) on the baseline RSNA and MAP (**A**), and on CSAR (**B**) in the PVN in control, OB, and OH rats. The values are mean ± SE. For each group, *n* = 6; * *p* < 0.05 versus saline; # *p* < 0.05 versus control; $ *p* < 0.05 versus OR; % *p* < 0.05 versus OB. SNP, sodium nitroprusside.

Compared with the microinjection of saline into PVN, the administration of N(ω)-propyl-l-arginine hydrochloride (PLA, 5 nmol, a nNOS inhibitor) in the PVN elicited significant increases only in baseline RSNA and MAP (Figure 3A), but not in CSAR (Figure 3B) in OB and OH rats. The increase in basal SNA in OB and OH rats was lower than in control and OR rats (*p* < 0.05). The peak of pressor and sympathoexcitatory responses due to the microinjection of PLA into the PVN was around 10–15 min after the injection, so CSAR induction was started 10 min after PLA infusion. The duration of PLA action in the PVN was around 30–40 min. 

### 2.6. Microinjection of Sodium Nitroprusside (SNP) into the PVN

The administration of sodium nitroprusside (SNP, 50 nmol, a NO donor) in the PVN elicited decreases in the baseline RSNA and MAP in the four groups, whereas a significant decrease of CSAR caused by SNP was observed only in the control and OR groups, not in OB and OH rats (Figure 3A,B) when compared with PVN microinjection of saline. The microinjection of PEG-SOD, DETC, PLA, and SNP outside the PVN did not change basal SNA and CSAR (data not shown). The peak of depressor and sympathoinhibitory responses due to the microinjection of SNP into the PVN was around 3–6 min after the injection, so CSAR induction was started 3 min after SNP infusion. The duration of SNP action in the PVN was around 10–15 min. Changes of the baseline RSNA and MAP caused by outside PVN microinjection of each chemical were shown in Table S3. 

### 2.7. Endogenous O_2_^−^ Level in the PVN

**Figure 4:**
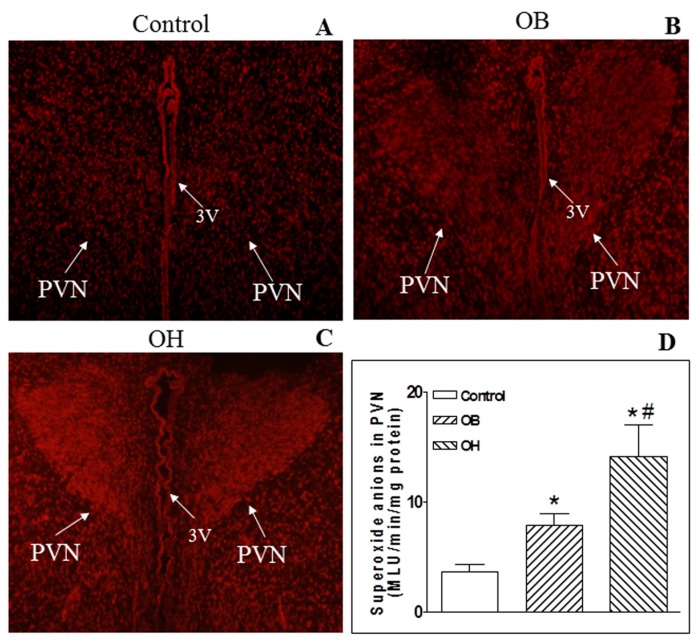
In situ O_2_^−^ in the PVN (**A**–**C**) and PVN O_2_^−^ levels (**D**) in control, OB, and OH rats. In situ O_2_^−^ was determined with a specific fluorogenic probe by the DHE method (*n* = 3 for each group), and arrows showed the microinjection sites. The levels of O_2_^−^ were determined by the lucigenin-derived chemiluminescence method. For each group, *n* = 6. The values are mean ± SE; * *p* < 0.05 versus control; # *p* < 0.05 versus OB. PVN: paraventricular nucleus; 3V: the third ventricle.

In situ detection of PVN O_2_^−^ by the dihydroethidium (DHE) method (a superoxide-sensitive dye staining technique) revealed a much higher fluorescent intensity in OB and OH rats compared with control rats (Figure 4A–C). The lucigenin-derived chemiluminescence method was used to detect the basal PVN O_2_^−^ production. We found that the level of O_2_^−^ in the PVN was higher in OB and OH rats than in control rats (*p* < 0.05 for each). However, OH rats had much more O_2_^−^ generation in the PVN than OB rats (*p* < 0.05, Figure 4D).

### 2.8. NADPH Oxidase and Its Activity in the PVN

**Figure 5:**
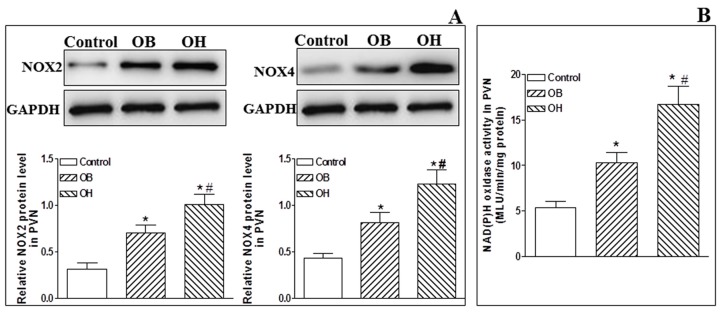
Protein expression levels of the NOX2 and NOX4 isoforms of NADPH oxidase (**A**) and NADPH oxidase activity (**B**) at the end of the 12th week in the PVN in control, OB, and OH rats. The values are mean ± SE; *n* = 4 for NOX2 and NOX4, *n* = 6 for PVN NADPH oxidase activity in each group; * *p* < 0.05 versus control; # *p* < 0.05 versus OB.

The protein expression levels of O_2_^−^-generating NADPH oxidase isoforms (NOX2 and NOX4) in the PVN were significantly upregulated in OB and OH rats when compared with control rats (*p* < 0.05 in all cases, Figure 5A), and they were much higher in OH rats. Moreover, we found the same tendency in NADPH oxidase activity as in NOX2 and NOX4 protein expression levels in OB and OH rats compared with control rats (*p* < 0.05 in all cases, Figure 5B). 

### 2.9. nNOS Protein Expression and Endogenous Nitric Oxide Metabolite (NOx) Level in the PVN

**Figure 6:**
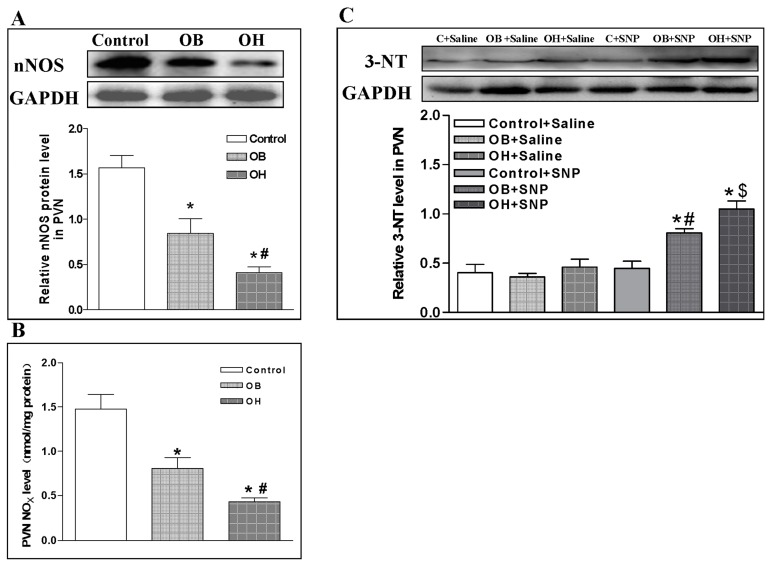
Relative values of nNOS protein expression levels (**A**), nitric oxide metabolite (NOx) levels (**B**), and 3-NT protein expression levels (**C**) after saline and SNP microinjection in the PVN at the end of the 12th week in control, OB, and OH rats. The values are mean ± SE; *n* = 4 for nNOS and 3-NT protein expression, *n* = 6 for PVN NOxlevels in each group; * *p* < 0.05 versuscontrol in (**A**,**B**); # *p* < 0.05 versus OB in Figure (**A**,**B**); * *p* < 0.05 versus Saline in (**C**); # *p* < 0.05 versus C + SNP in (**C**); $ *p* < 0.05 versus OB + SNP in (**C**). 3-NT: 3-nitrotyrosine; C: control.

There was a lower level of nNOS protein in OB rats than in control rats, and an even lower level in OH rats than in OB rats (*p* < 0.05 in all cases, Figure 6A). NOx level is widely used as an index of NO level [24]. The endogenous NOx content in the PVN was also lower in OB rats than in control rats, and it was the lowest in OH rats among the three groups (*p* < 0.05 in all cases, Figure 6B).

### 2.10. Effect of SNP in the PVN on 3-Nitrotyrosine (3-NT) Protein Expression

The molecule 3-nitrotyrosine (3-NT) is a product of protein tyrosine nitration resulting from oxidative damage of proteins by peroxynitrite. There was no significant difference in 3-NT levels after PVN treatment with saline in control, OB, and OH rats, but 3-NT levels were significantly increased in the PVN after PVN treatment with SNP in OB and OH rats compared with control animals (*p* < 0.05 in all cases, Figure 6C).

### 2.11. Plasma and PVN Ang II Levels and AT1R Protein Expression in the PVN

**Figure 7:**
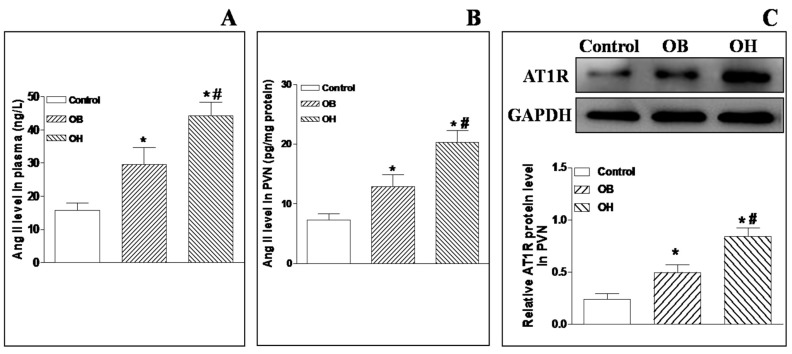
Plasma Ang II levels (**A**), PVN Ang II levels (**B**), AT1R protein expression (**C**) in control, OB, and OH rats. The values are mean ± SE; *n* = 4 for AT1R protein expression, *n* = 6 for plasma Ang II levels and PVN Ang II levels, in each group; * *p* < 0.05 versus control; # *p* < 0.05 versus OB. AT1R: Ang II type-1 receptor.

Compared with control rats, there were significant higher levels of plasma and PVN Ang II in OB and OH rats, and the levels were much higher in OH rats than OB rats (*p* < 0.05 in all cases, Figure 7A,B). In addition, Ang II type-1 receptor (AT1R) protein expression in the PVN was significantly increased in OB and OH rats compared with control rats (*p* < 0.05 in all cases, Figure 7C). 

### 2.12. Effects of the Pretreatment with Saline, Losartan, PEG-SOD, or SNP in the PVN on CSAR Response to PVN Microinjection of Ang II

**Figure 8:**
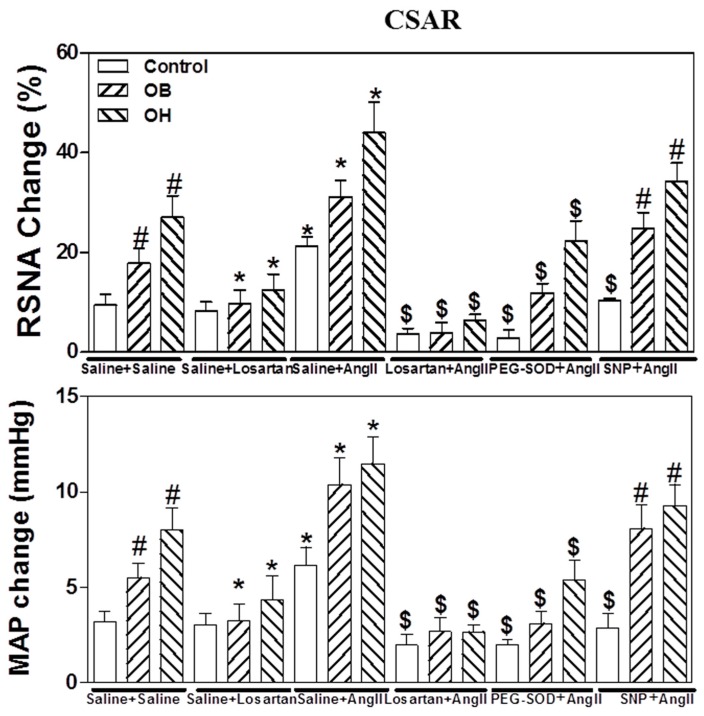
The effects of the pretreatments consisting in PVN microinjection of saline, losartan (10 nmol), PEG-SOD (5 units), or sodium nitroprusside (SNP, 50 nmol) on CSAR response to PVN microinjection of Ang II (0.3 nmol). For each group, *n* = 6. The values are mean ± SE; * *p* < 0.05 versus saline. # *p* < 0.05 versus control; $ *p* < 0.05 versus Ang II.

The microinjection of Ang II (0.3 nmol) into the PVN significantly potentiated basal SNA and CSAR in control, OR, OB, and OH rats after pretreatment with saline in the PVN. The effects of Ang II on CSAR were much stronger in OB and OH rats than in control and OR rats (*p* < 0.05 in all cases, Figure 8). The microinjection of losartan or PEG-SOD into the PVN markedly inhibited the enhanced basal SNA and CSAR responses to PVN microinjection of Ang II in the four groups. However, the microinjection of SNP into the PVN did not significantly attenuate the enhanced basal SNA and CSAR response to PVN microinjection of Ang II in OB and OH rats compared with control and OR rats (Figure 8).

## 3. Discussion

The results of this study indicate that, in obese rats, there was an increase in PVN O_2_^−^, Ang II, AT1R, and NADPH oxidase levels, and a decrease in nNOS protein levels and NO content in the PVN. In addition, CSAR was markedly elevated, and it promoted a significant increase of plasma NE levels in OB and OH rats. The enhanced CSAR was reduced by PVN application of PEG-SOD (an analogue of endogenous superoxide dismutase) in all groups, which was more obvious in OB and OH rats. CSAR enhancement was significantly reduced by the NO donor SNP only in control and OR rats, not in OB and OH rats. Moreover, CSAR was further strengthened by the superoxide dismutase inhibitor DETC in all groups, and this was also more obvious in OB and OH rats. However, the nNOS inhibitor PLA administrated in the PVN elicited a marked increase in basal SNA and CSAR in control and OR rats, whereas its effects on CSAR in OB and OH rats was not significant when compared with control rats. Finally, the enhanced CSAR response to exogenous Ang II in the PVN was markedly attenuated by PVN infusion of losartan and PEG-SOD, but not of SNP. These data suggest that the enhanced CSAR, mediated by an increase of endogenous O_2_^−^ and a lack of NO within the PVN, may contribute to the elevated SNA and blood pressure observed in obesity. 

The enhanced SNA plays an important role in obesity-related cardiovascular diseases [25,26,27,28]. Many studies have explored the potential mechanisms involved in increased SNA in obesity, including central mechanisms. For instance, our group has shown that there was a higher plasma NE level in obese rats, and an even higher level in obesity-related hypertensive rats, which indicates a sympathetic hyperactivity during obesity [14]. CSAR, a sympathoexcitatory reflex, contributes to sympathetic overdrive and the elevation of blood pressure in animal models of CHF, hypertension, and diabetes [6,7,9]. In this study, the CAP-induced CSAR response was enhanced in OB rats, and this effect was much stronger in OH rats. Moreover, the CSAR response to CAP obviously promoted the increase of plasma NE levels in OB and OH rats, suggesting that the enhanced CSAR may activate sympathetic outflow in obesity. 

It is now widely recognized that neuronal production of O_2_^−^ and NO influences cardiovascular functions through neuromodulatory actions within the autonomic nervous system. PVN is a critical area in the O_2_^−^ and NO generation and in the maintenance of sympathetic control of the cardiovascular system [5,18,19,20,21,22,29]. In this study, there was an increased O_2_^−^ level and a decreased NO generation in the PVN of OB and OH rats indicating that the changes of O_2_^−^ and NO production may be partially responsible for the enhanced CSAR and sympathetic overdrive during obesity. Previous studies have suggested that PVN O_2_^−^ and NO can participate in the regulation of CSAR in renovascular hypertensive rats [8,18,23,30,31]. In the present study, we indeed observed a greater decrease or increase in CSAR amplitude upon the removal of O_2_^−^ with an analogue of endogenous superoxide dismutase for scavenging O_2_^−^ (PEG-SOD), or by using the superoxide dismutase inhibitor DETC in the PVN in OB and OH rats, respectively. However, a more significant increased or decreased CSAR response to an inhibitor for nNOS or NO donor in the PVN was only found in control rats, not in obese rats. These results suggest that O_2_^−^ and NO in the PVN are involved in a tonic excitation or inhibition, respectively, but NO does not exert enough tonic inhibition in the PVN for the enhanced CSAR in obesity, which may be related to the reduced NO level or bioavailability in the PVN. In fact, an imbalance in O_2_^−^ (increase) and NO (decrease) promotes the excitation of the sympathetic nervous system, and this mechanism seems to be involved in the pathogenesis of neurogenic aspects of hypertension [8,18,23,30,31].Therefore, the imbalance of O_2_^−^ and NO generation in the PVN may lead to the enhancement of CSAR contributing to sympathetic overactivity during obesity.

It is well known that the excessive formation of O_2_^−^ in hypertension may be associated with NADPH oxidase, Ang II, and AT1 receptor. PVN O_2_^−^ produced by Ang II has been indicated to mediate the central Ang II-induced sympathoexcitatory action including CSAR [8,18,23,30,31]. Moreover, NADPH oxidase in PVN could contribute to the effects of Ang II on CSAR, and Ang II, via AT1R, increased NADPH oxidase activity that is responsible for O_2_^−^ generation [8,18,23,30,31]. In our study, there was a marked increase in the PVN Ang II, AT1 receptor, NADPH oxidase, and O_2_^−^ levels in OB and OH rats. Moreover, a pretreatment with losartan or PEG-SOD within the PVN obviously attenuated the effects of Ang II on CSAR in normal and obese rats. Therefore, it is possible that the elevated levels of Ang II, AT1R, and NADPH oxidase in the PVN may promote the excessive production of O_2_^−^ in obesity, which is responsible for the augmented effects of Ang II in the PVN on CSAR in obese rats.

NO, a sympathoinhibitory neurotransmitter in the CNS, primarily derives from nNOS, a major isozyme of NOS in the CNS, including the PVN [32,33]. Intracerebroventricular infusions of NOS inhibitors lead to hypertensive effects [34]. The same results were observed in the nucleus tractus solitaries (NTS) [35], whereas there was a decrease in blood pressure caused by NOS inhibitors in the caudal ventrolateral medulla (CVLM) [36]. The inhibition of NO synthesis in the (rostral ventrolateral medulla) RVLM and the NTS causes the attenuation of the baroreflex, but the impairment of NO synthesis within the PVN contributes to an augmented peripheral chemoreflex and a subsequent elevation of the sympathetic activity which is characteristic of heart failure [37]. Increased NO in the RVLM improves the impaired baroreflex control in stroke-prone spontaneous hypertension rats [38]. In the PVN, endogenous NO within the PVN is involved in the regulation of sympathetic outflow, and it could exert an inhibitory effect on neurons involving the regulation of arterial pressure and SNA [5,21,22,23]. Rats with CHF or renovascular hypertension had a decreased nNOS expression, and, consequently, decreased NO in the PVN [39,40], indicating a loss of the regulation of the sympathetic tone. Indeed, we also found that there were much lower nNOS protein expression and NO content in the PVN of obese rats, which further indicates that the reduction of PVN NO does not sufficiently suppress the tonic excitation in the PVN in obesity. Consistent with this result, we found that PLA, a selective inhibitor of nNOS, and SNP, a NO donor, in the PVN caused more significant elevation and reduction in CSAR and SNA in normal rats, but their effects were smaller in obese rats. This result suggests that NO is an important mediator of SNA in the PVN in obesity, and the increase or decrease in obese rats indicate that CSAR is under less tonic inhibition from PVN NO in obese rats than in control rats. 

The NO bioavailability in a given tissue depends not only on the rate of its production, but also on the rate of its inactivation by O_2_^−^ (NO + O_2_^−^ → ONOO^−^). It was demonstrated that O_2_^−^ can react with and inactivate NO, and thereby modulate its bioavailability [41], which may be involved in the pathogenesis of hypertension in hypertensive rats [42]. Therefore, in this study, it is possible that excessive O_2_^−^ reacts with NO resulting in functional NO deficiency in the PVN of obese rats. We also measured the 3-NT level reflecting the ONOO^−^ level and observed that there was no significant change in 3-NT levels in obese rats compared to control rats. However, an infusion of an NO donor SNP in the PVN caused a significant increase in 3-NT levels in obese rats but not in control rats, which indicates that NO itself was very low in the obesity state, and an exogenous increase of NO did not adequately inhibit CSAR, because NO may be partly deactivated by the interaction with O_2_^−^. In addition, in obese rats, scavenging O_2_^−^ with PEG-SOD abolished Ang II-induced CSAR enhancement, but the NO donor SNP did not, which further implies that NO in the obesity state cannot exert an effective inhibition of CSAR. 

**Figure 9:**
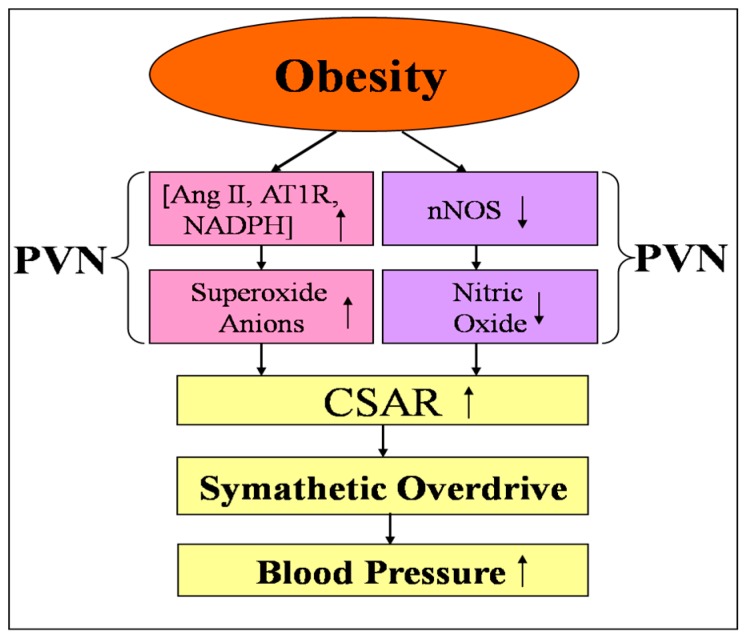
Schematic diagram showing the possible mechanisms in the paraventricular nucleus (PVN) involved in the regulation of the enhanced cardiac sympathetic afferent reflex (CSAR) and sympathetic outflow in the obesity state. The up arrows indicate significant increase in Ang II, AT1R, NADPH, CSAR and blood pressure, and the down arrows indicate significant decrease in nNOS and nitric oxide. Ang II: angiotensin II; AT1R: Ang II type-1 receptor; NADPH: nicotinamide adenine dinucleotide phosphate; nNOS: neural nitric oxide synthase.

The present results show that CSAR is enhanced in OB and OH, and the enhanced CSAR is involved in sympathetic activation. The PVN modulates the enhanced CSAR and sympathetic outflow in obesity hypertension. This discovery shows an important role of the enhanced CSAR in sympathetic activation and hypertension in obesity. Furthermore, our results indicate that both NO and O_2_^−^ in the PVN participate in the regulation of enhanced CSAR (Figure 9). Interventions on the enhanced CSAR aimed at increasing NO and decreasing O_2_^−^ levels in the PVN may be a strategy for attenuating hypertension in obesity. 

## 4. Materials and Methods

### 4.1. Experimental Design

All rats received a control diet or HFD feeding for 12 weeks. Body weight (BW) and systolic blood pressure (SBP) were assessed in a conscious state. At the end of the 12th week, acute experiments were carried out. Histological identifications of PVN injection sites were performed in all the rats subjected to the corresponding injections. An experiment graphical scheme is shown in Figure S1.

Experiment 1: The levels of plasma NE, Ang II (*n* = 6 for each), PVN Ang II (*n* = 6 for each), NO (*n* = 6 for each), superoxide anions, NADPH oxidase activity (*n* = 3 for each group for in situ detection of superoxide anions, and *n* = 6 for each group for both superoxide anions levels and NADPH oxidase activity measurements), nNOS, NADPH oxidase subunits NOX2 and NOX4, 3-NT, and AT1R (*n* = 4 for each group) were determined in control, OB, and OH rats.

Experiment 2: CSAR was evaluated by the RSNA and MAP responses to CAP in control, OR, OB, and OH. The changes of plasma NE level caused by the application of CAP (1 nmol) to induce CSAR were determined in control, OR, OB, and OH rats. Each rat was subjected to the epicardial surface of left ventricle application of vehicle, CAP, and vehicle, in turn. The intervals between applications were at least 30 min for complete recovery. The blood samples were collected from the carotid artery for NE measurement 1 min after each application (*n* = 6 for each group).

Experiment 3: The effects of PVN microinjection of saline, PEG-SOD (5 units), diethyldithiocarbamic acid (DETC, 10 nmol), PLA (5 nmol), and sodium nitroprusside (SNP, 50 nmol) on the CSAR and on the baseline RSNA and MAP were determined in five groups of control rats, five groups of OB rats, and five groups of OH rats (*n* = 6 for each). CSAR was determined 10 min after the PVN microinjection of saline, PEG-SOD, and PLA, 3 min after DETC, and 1 min after SNP, and the microinjection volume in the PVN was 50 nL for each of the tested substances.

Experiment 4: The effects of pretreatments consisting in PVN microinjections of saline, losartan (10 nmol), PEG-SOD (5 units), or SNP (50 nmol) on CSAR and on the baseline RSNA and MAP responses to PVN microinjection of Ang II (0.3 nmol) were investigated in six groups of control rats, six groups of OB rats, and six groups of OH rats (*n* = 6 for each group). The PVN microinjection of Ang II was carried out 10 min after pretreatment with lorsartan, 20 min after PEG-SOD (5 units), and 1 min after SNP. The CSAR was determined 2 min after Ang II microinjection, and the microinjection volume in the bilateral PVN was 100 nL for each of the tested substances.

### 4.2. Animals

Male Sprague Dawley rats (10–12 weeks old) weighing between 300–350 g were randomly divided into two groups; one group received a control diet (12% kcal as fat: 12% fat, 60% carbohydrate, 28% protein, TROPHIC Animal Feed High-tech Co. Ltd., Nantong, China) and the other received a high-fat diet (HFD, 42% kcal as fat: 42% fat, 43% carbohydrate, 15% protein, TROPHIC Animal Feed High-tech Co. Ltd., Nantong, China) for 12 weeks. All rats were given food and water and were housed in a temperature- and humidity-controlled room with a 12h light–dark cycle. After 12 weeks, the rats consuming the HFD were ranked based on weight gain. The rats with higher weight gains and a systolic blood pressure (SBP) ≥ 150 mm Hg were referred to as obesity-related hypertensive rats (OH group), and the rats with higher weight gains and a lower SBP were referred to as obese non-hypertensive rats (OB group, SBP < 150 mm Hg). The rats receiving the control diet were used as a control group. The rats with the HFD diet without higher weight gains and an SBP < 150 mm Hg were used as the obesity resistant (OR) group. The experimental procedures were approved by the Experimental Animal Care and Use Committee of Nanjing Medical University (SYXK2013-0016, 14 May 2013) and complied with the Guide for the Care and Use of Laboratory Animals (NIH publication, Eighth edition, 2011).

### 4.3. Measurement of Body Weight and SBP

At the end of the 12th week, the body weight (BW) of rats was measured in a conscious state. A noninvasive computerized tail-cuff system (NIBP, AD Instruments, Sydney, Australia) was used for the measurement of SBP in the tail artery. In order to detect the pulsation of the tail artery and obtain a steady pulse level, the rats were warmed for about 20 min at 28 °C. Moreover, the rats were trained by measuring SBP daily for at least 10 days before performing the acute experiment, to minimize SBP fluctuations, and SBP was determined by averaging 10 measurements [14].

### 4.4. Blood and PVN Sample Preparation

The changes of the plasma NE levels after the administration of CAP used to induce CSAR were determined in anesthetized rats. Blood samples of about 250 μL were collected from the carotid artery for NE measurements 1 min after CAP application. The blood, about 1 mL, from the tail top of conscious rats, was collected for measuring the levels of glucose, insulin, triglyceride, and Ang II at the end of 12 weeks. For some parameters detected in the PVN such as O_2_^−^ and NO level, each rat was anaesthetized with an overdose of sodium pentobarbital by intraperitoneal injection, and then their brains were quickly removed and frozen in liquid nitrogen. Finally, the plasma and brains were stored at −80 °C until used. Coronal sections of the brain were obtained with a cryostat microtome (Leica CM1900-1-1, Wetzlar, Hessen, Germany) at the PVN level [21,22,23]. 

### 4.5. Measurement of Plasma Glucose, Insulin, Cholesterol, and Triglyceride Levels

About 1.5 mL of blood was collected from the tail vein at the end of 12 weeks. All rats were fasted overnight, then fasting plasma glucose (using the glucose oxidase method and a kit from Jiancheng Bioengineering, Nanjing, China), fasting plasma insulin (using the enzyme-linked immunoassay (Elisa) method and a kit from RayBiotech, Inc., Norcross, GA, USA), cholesterol, and triglyceride (using a commercial colorimetric assay and kits from Jiancheng Bioengineering, Nanjing, China) were measured in control, OB, and OH rats, according to the manufacturer’s instructions.

### 4.6. Measurement of Plasma NE and Ang II Levels, and PVN Ang II Level 

The collected PVN tissue samples were homogenized by a Branson 250 sonicator (Branson Ultrasonics, Danbury, CT, USA), then total proteins in the homogenate supernatant were extracted and measured. The reactions were performed in 96-well plates with antibodies specific for rat NE or Ang II. A standard diluent buffer and the samples were added into plates, incubated overnight, and washed four times. A horseradish peroxidase-conjugated solution was added and then washed out four times. At last, the reactions were stopped with a stop solution, and the final solution was read at 450 nm using a microplate reader (ELX800, BioTek, Winooski, VT, USA). The kits for the determination of NE and Ang II levels were from USCN Business Co., Ltd. (Wuhan, China).

### 4.7. In Situ Detection of Superoxide Anions

The specific fluorogenic probe dihydroethidium (DHE) method was used to determine in situ superoxide anions in the PVN. The brains were coronally sectioned (30 μm) and the PVN regions were placed onto chilled microscope slides. The sections were thawed and rehydrated using phosphate-buffered saline, then incubated with DHE (1 μmol/L) in the dark. DHE fluorescence was visualized by using confocal microscopy (Zeiss LSM 510, Carl Zeiss, Jena, Thuringia, Germany) [8].

### 4.8. Measurement of Superoxide Anions

The level of superoxide anions was detected using the lucigenin-derived chemiluminescence method in the PVN [8]. Dark-adapted lucigenin (5 μM) reacts with superoxide anions resulting in photon emission which can be detected by a luminometer (20/20n, Turner, Sunnyvale, CA, USA) once every minute for 10 min. The data representing the superoxide anions level were expressed as the mean light units (MLU) per minute per milligram of protein.

### 4.9. Measurement of NADPH Oxidase Activity

The activity of PVN NADPH oxidase was determined by the enhanced lucigenin chemiluminescence method [27]. Briefly, NADPH (100 μM) used as a substrate, reacts with NADPH oxidase in the medium to generate superoxide anions which can react with lucigenin (5 μM) to produce light emission. A luminometer (20/20n, Turner, CA, USA) was used to detect the light emission once every minute for 10 min. The data representing the NADPH oxidase activity were expressed as the (MLU) per minute per milligram of protein.

### 4.10. Measurement of NO Metabolites (NOx) Level in the PVN

NO generation in the PVN was determined by the determination of the concentration of its stable metabolites nitrates and nitrites using a Nitrate-Nitrite Colorimetric Assay Kit (Cayman Chemical Company, Ann Arbor, MI, USA). According to manufacturer’s instructions, the total proteins were extracted from the homogenate supernatant, the supernatant was ultrafiltered, and then an eluant was used to evaluate the NOx level. The absorbance of the samples was measured by using a microreader in the wavelength of 540 nm [27].

### 4.11. Measurement of nNOS, NOX2, NOX4, AT1R, and 3-NT Protein Expression Levels

The protein expression levels of nNOS, NOX2, NOX4, AT1R, and 3-NT in the PVN were measured as previously reported [27]. Simply, total PVN proteins in the homogenate were extracted and measured. Western blot analyses were carried out with the use of rabbit polyclonal antibodies against nNOS (Cell Signaling Technology, Danvers, MA, USA), NOX2, NOX4, 3-NT (Abcam, Burlingame, CA, USA), AT1R (Santa Cruz Biotechnology, Santa Cruz, CA, USA), and GAPDH (Bioworld Technology, Louis Park, MN, USA) as the primary antibodies. The peroxidase-conjugated goat anti-rabbit secondary antibody (Santa Cruz Biotechnology, Santa Cruz, CA, USA) was used as a secondary antibody. The levels of nNOS, NOX2, NOX4, 3-NT, and AT1R were expressed as the ratio to GAPDH protein levels.

### 4.12. General Procedures of the Acute Experiment

After 12 weeks, the acute experiment was carried out. The rats were anesthetized with urethane (800 mg/kg) and α-chloralose (40 mg/kg) intraperitoneally. The trachea and carotid artery were exposed by a midline incision, then the trachea was cannulated and connected with a rodent ventilator (Model 683, Harvard Apparatus Inc., Holliston, MA, USA), and the right carotid artery was cannulated to record the mean arterial pressure (MAP) and heart rate (HR). The renal sympathetic nerve activity (RSNA), and MAP were simultaneously recorded with a PowerLab data acquisition system (8/35, ADInstruments, Sydney, Australia). Supplemental doses of anesthetic agents were used to maintain an adequate depth of anesthesia during the experiment.

### 4.13. Vagotomy and Baroreceptor Denervation

The operations of vagotomy and baroreceptor denervation were made on rats in order to minimize the confounding role of the baroreflex in SNA and BP, as previously reported [23]. The baroreceptor denervation was assumed to be successful if HR was altered less than 5 beats/min after the intravenous injection of phenylephrine (20 μg/kg).

### 4.14. RSNA Recording

RSNA was used to assess the dynamic alterations of sympathetic outflow. Simply, the left renal sympathetic nerve was isolated through a retroperitoneal incision and cut distally to abolish its afferent activity. The nerve was placed on a pair of silver electrodes and immersed in mineral oil. The RSNA was amplified with an alternating current/direct current (AC/DC) differential amplifier (Model 3000; A-M System, Washington, DC, USA) and filtered with a band-pass between 10 and 3000 Hz. The signals were integrated at the time constant of 100 ms. The baseline noise of the recorded signals was measured after sectioning the central end of the nerve at the end of the experiment, and subtracted from the integrated RSNA data. The RSNA change was the percent change from the baseline. Baseline RSNA and MAP were determined by averaging 1 min of their maximal responses after the PVN microinjection. CSAR was evaluated by averaging 1 min of the maximal RSNA and MAP responses to CAP within the time range from 30 ms to 5 min after the application of CAP, and the RSNA change was the percent change of the values before CAP. 

### 4.15. CSAR Induction and Evaluation

A limited thoracotomy was made, and the pericardium was removed to expose the heart. The CSAR was induced by application of a piece of filter paper (3 × 3 mm) containing capsaicin (CAP, 1 nmol in 2 μL) to the epicardial surface of the left ventricle of the rats for 1 min. The epicardium was rinsed with 10 mL of warm normal saline (38 °C) for three times after removing the filter paper. The CSAR was assessed by the responses of the RSNA and MAP to CAP, as previously reported [23].

### 4.16. PVN Microinjection

The stereotaxic coordinates for PVN were 1.8 mm caudal from bregma, 0.4 mm lateral to midline, and 7.9 mm ventral to dorsal surface, referring to the atlas of the rat brain. To locate the PVN, each rat was fixed in a stereotaxic frame (Stoelting; Chicago, IL, USA), then a microinjection volume of 50 nL for each side of PVN was applied by two glass micropipettes (50 μm tip diameter); the bilateral microinjections of chemicals were finished within 1 min. At the end of the experiment, the same volume of Evans Blue was injected into the microinjection sites for histological identification of the PVN. A representative photo of microinjection sites in the PVN evaluated by Evans blue (50 nL) diffusion was shown in Figure S2. The data would be excluded from data analysis if the microinjection sites were outside the PVN. A representative photo of the microinjection sites in the PVN evaluated by 50 nL of Evans blue diffusion is shown in Figure S1. 

### 4.17. Chemicals

Capsaicin (CAP), PEG-SOD, NADPH (100 μM), lucigenin (5 μM), DETC, N(ω)-propyl-l-arginine hydrochloride (PLA), SNP, and Ang II were obtained from Sigma Chemical (St. Louis, MO, USA). The doses of CAP (1 nmol), PEG-SOD (5 units), diethyldithiocarbamic acid (DETC, 10 nmol), PLA (5 nmol), SNP (50 nmol), and Ang II (0.3 nmol) that were applied in the experiments, were chosen with reference to our preliminary studies and published papers [27].

### 4.18. Statistical Analysis

All data were analyzed using SPSS version 17.0 (SPSS Inc., Chicago, IL, USA). There were 341 obesity-prone (OP) rats (72.9%) in 468 high-fat diet-fed rats. In these OP rats, 226 rats (66.3%) developed hypertension, and 115 rats (33.7%) maintained a normal blood pressure level. The comparisons between two observations in each animal were analyzed by Student’s *t* test. The differences between groups were determined with a one-way or two-way ANOVA followed by the Bonferroni test for post hoc analysis of significance. All data were expressed as means ± SE. A value of *p* < 0.05 was considered statistically significant.

## Supplementary Materials

Supplementary materials can be found at www.mdpi.com/1422-0067/19/1/59/s1.
