# Supplementary material for: Superoxide Anions and NO in the Paraventricular Nucleus Modulate the Cardiac Sympathetic Afferent Reflex in Obese Rats
Source: Int J Mol Sci. 2017 Dec 27;19(1):59. doi: 10.3390/ijms19010059 (PMC5796009; doi:10.3390/ijms19010059)
Supplement: Supplementary file 1 [file ijms-19-00059-s001.pdf]

ONLINE SUPPLEMENT

Superoxide anions and NO in paraventricular nucleus modulate cardiac sympathetic afferent reflex in obese rats

Qing-Bo Lu[1], Jing sun[1], Ying Kang[1], Hai-Jian Sun[1], Lei Ding[1], Hui-Shan Wang[2], Yuan Wang[2], Guo-Qing Zhu[1], Ye-Bo Zhou[1,*]

[1] Key Laboratory of Cardiovascular Disease and Molecular Intervention, Department of Physiology, Nanjing Medical University, Nanjing 210029, China;

[2] Department of Pediatrics, the Fourth Clinical Medical College of Nanjing Medical University, Nanjing 210029, China.

***Address for correspondence:**

Ye-Bo Zhou, M.D., Ph.D.
Key Laboratory of Cardiovascular Disease and Molecular Intervention, Department of Physiology, Nanjing Medical University,
101 Longmian Road, Nanjing 211166, China
Tel: +86-25-86862885
Fax: +86-25-86862885
E-Mail: zhouyebo666@njmu.edu.cn

Deleted: 163.com

Supplemental Tables.

Table S1. Metabolic parameters, anatomic data, systolic blood pressure, mean arterial pressure, and heart rate in control, OR, OB and OH rats after 12 wks of diet.

	Control	OR	OB	OH
N	115	60	115	115
BW (g)	506±5	510±6	613±6*	618±5*
Plasma glucose (mg/dl)	129±3	132±2	143±3	140±4
Plasma insulin (ng/ml)	1.51±0.03	1.56±0.04	2.86±0.04*	2.87±0.05*
Plasma cholesterol (mg/dl)	41.9±0.3	44.8±0.4	55.7±0.4*	57.3±0.6*
Plasma triglyceride (mg/dl)	65.5±0.8	69.9±0.7	85.8±0.8*	86.1±0.9*
HW (mg)	1651±18	1667±19	2128±28*	2197±23*
HW/BW (mg/g)	3.30±0.06	3.06±0.05	3.43±0.07	3.44±0.06
SBP (mm Hg)	124±2	126±3	132±3	171±4*, $^{\#,\%}$
MAP (mm Hg)	93.4±1.7	92.7±1.8	96.5±1.9	131.7±2.1*, $^{\#,\%}$
HR (bpm)	359±6	363±5	371±6	423±7*, $^{\#,\%}$
Sum of WAT mass (g)	26.6±0.4	27.9±0.5	53.1±1.1*	54.6±1.2*

OR, obesity resistant, OB: obesity without hypertension; OH: obesity-related hypertension; BW, body weight; HW, heart weight; SBP, systolic blood pressure; MAP, mean arterial pressure; HR, heart rate; WAT, white adipose tissue; Sum of WAT mass includes inguinal, retroperitoneal, epididymal and mesenteric WAT mass. Values are

mean±SE. *$P<0.05$ vs. Control. #$P<0.05$ vs. OB. %$P<0.05$ vs. OR. SBP was measured under conscious state, and MAP and HR were determined under anesthesia.

Table S2. Changes of the baseline RSNA and MAP caused by PVN microinjection of saline, PEG-SOD (5 units), DETC (10 nmol), PLA (5 nmol) and SNP (50 nmol) in control, OR, OB and OH rats, respectively.

	Control	OR	OB	OH
Saline: ΔRSNA	0.48±1.04	0.20±0.88	0.30±1.23	0.28±0.77
Saline: ΔMAP	0.64±0.56	0.95±0.83	0.78±1.12	0.17±0.79
PEG-SOD: ΔRSNA	-4.63±0.63*	-4.92±0.72*	-12.08±0.94*$^{, \#,\%}$	-21.37±2.71*$^{, \#,\%, \$}$
PEG-SOD: ΔMAP :	-3.07±0.68*	-3.13±0.86*	-5.67±0.80*	-9.00±1.13*$^{, \$}$
DETC: ΔRSNA	11.40±1.46*	10.10±0.68*	17.55±1.58*$^{, \#,\%}$	26.08±3.22*$^{, \#,\%, \$}$
DETC: ΔMAP	5.67±0.88*	5.50±1.18*	8.00±1.71*	11.50±1.31*$^{, \$}$
PLA: ΔRSNA	10.17±0.8	8.98±2.20*	5.65±0.55*$^{, \#,\%}$	2.90±0.52*$^{, \#,\% \$}$
PLA: ΔMAP	8.75±0.99*	8.48±0.88*	5.33±0.49*$^{, \#,\%}$	2.28±0.50$^{\#, \%,\$}$
SNP: ΔRSNA	-32.77±2.75*	-32.58±3.10*	-18.42±2.14*$^{, \#,\%}$	-9.32±1.72*$^{, \#, \%,\$}$
SNP: ΔMAP	-13.83±1.42*	-14.33±1.56*	-9.00±0.82*$^{, \#,\%}$	-4.67±0.88*$^{, \#,\%, \$}$

Values are mean±SE. n=6 for each group. *P<0.05 vs. Saline. $^{\#}$P<0.05 vs. Control. $^{\%}$P<0.05 vs. OR. $^{\$}$P<0.05 vs. OB. PVN: paraventricular nucleus; CSAR: cardiac sympathetic afferent reflex; RSNA: renal sympathetic nerve activity; MAP: mean artery pressure; OR, obesity resistant; OB: obesity without hypertension; OH: obesity-related hypertension; PEG-SOD: superoxide scavenger; DETC: superoxide dismutase inhibitor; PLA: nNOS inhibitor; SNP: NO donor. The microinjection volume in bilateral PVN was

100 nL for each substances described. Baseline RSNA and MAP were determined by averaging 1 min of its maximal responses after the PVN microinjection.

Table S3. Changes of the baseline RSNA and MAP caused by outside PVN microinjection of saline, PEG-SOD (5 units), DETC (10 nmol), PLA (5 nmol), SNP (50 nmol), Ang II (0.3 nmol) and Losartan (10 nmol) in control, OR, OB and OH rats, respectively.

	Control	OR	OB	OH
Saline: ΔRSNA	2.56	3.25	None	None
Saline: ΔMAP	1.64	2.17	None	None
PEG-SOD: ΔRSNA	-1.48	None	None	None
PEG-SOD:ΔMAP	-0.96	None	None	None
DETC: ΔRSNA	2.48	None	None	None
DETC:ΔMAP	1.06	None	None	None
PLA: ΔRSNA	None	-1.20	None	1.38
PLA: ΔMAP	None	-2.37	None	-1.26
SNP: ΔRSNA	None	None	3.30	None
SNP: ΔMAP	None	None	2.6	None
Ang II: ΔRSNA	None	None	2.58	1.26
Ang II: ΔMAP	None	None	1.69	2.55
Losartan: ΔRSNA	None	None	-1.31	3.29
Losartan: ΔMAP	None	None	1.57	1.07

In this study, we had 16 rats missed the PVN, 5 rats in Control group (Saline 2 rats,

PEG-SOD 1 rat, and DETC 2 rats), 2 rats in OR group (Saline 1 rat and PLA 1 rat), 5 rats in OB group (SNP 2 rats, Ang II 2 rats, and Lorsatan 1 rat), and 4 rats in OH group (PLA 1 rat, Lorsatan 1 rat, and Ang II 2 rats). PVN: paraventricular nucleus; RSNA: renal sympathetic nerve activity; MAP: mean artery pressure; OR, obesity resistant, OB: obesity without hypertension; OH: obesity-related hypertension; PEG-SOD: superoxide scavenger; DETC: superoxide dismutase inhibitor; PLA: nNOS inhibitor; SNP: NO donor; Ang II: angiotensin II. The microinjection volume outside PVN was 100 nL for each substances described. Baseline RSNA and MAP were determined by averaging 1 min of its maximal responses after microinjection.

Supplemental Figure

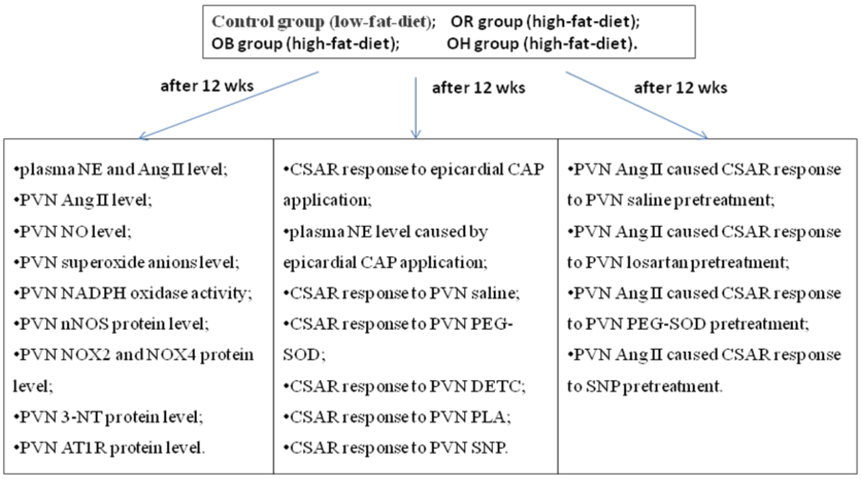

Figure S1. An experiment graphical scheme for study protocol. Rats were divided into 3 or 4 groups and fed low-fat-diet or high-fat-diet for12 wks. PVN: paraventricular nucleus; RSNA: renal sympathetic nerve activity; MAP: mean artery pressure; OR, obesity resistant, OB: obesity without hypertension; OH: obesity-related hypertension; PEG-SOD: superoxide scavenger; DETC: superoxide dismutase inhibitor; PLA: nNOS inhibitor; SNP: NO donor; Ang II: angiotensin II. At the end of the 12th week, some rats were sacrificed, and plasma and PVN tissues were collected for the examination of norepinephrine (NE), Ang II, nitric oxide (NO) and superoxide anions levels, nicotinamide adenine dinucleotide phosphate (NADPH) oxidase activity, and neural NO synthase (nNOS), NADPH oxidase subunits NOX2 and NOX4, nitrotyrosine (3-NT), and Ang II type 1 receptor (AT1R) protein levels. Acute experiments were carried out for the research of CSAR response to chemical reagents.

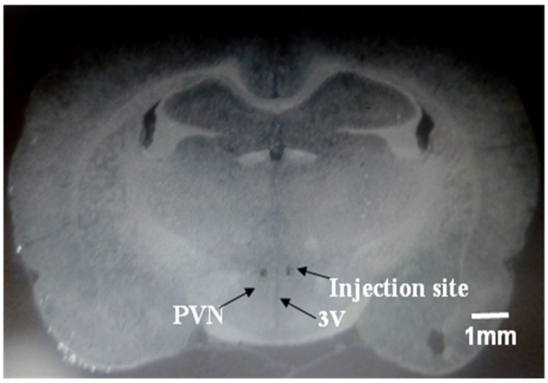

Figure S2. A representative photo of microinjection sites in the PVN evaluated by Evans blue (50 nL) diffusion. Arrows indicate the microinjection sites, PVN: paraventricular nucleus; 3V: the third ventricle.
